# Screening and testing for tuberculosis among the HIV-infected: outcomes from a large HIV programme in western Kenya

**DOI:** 10.1186/s12889-018-6334-4

**Published:** 2019-01-08

**Authors:** Philip Owiti, Dickens Onyango, Robina Momanyi, Anthony D. Harries

**Affiliations:** 1 Academic Model Providing Access to Healthcare (AMPATH), Eldoret, Kenya; 2 International Union Against Tuberculosis and Lung Disease, Paris, France; 3 National Tuberculosis, Leprosy and Lung Disease Programme, Nairobi, Kenya; 4 County Department of Health, Kisumu, Kenya; 5 Moi Teaching and Refferal Hospital, Eldoret, Kenya; 6 London School of Hygiene and Tropical Medicine, London, UK

**Keywords:** Active case finding, Intensified case finding, Screening, Tuberculosis, HIV, Quality care

## Abstract

**Background:**

People living with HIV (PLHIV) are at increased risk of tuberculosis (TB). TB is also the leading opportunistic infection contributing to about one-third of deaths in this population. The World Health Organization recommends regular screening for TB in PLHIV. Those identified to have any TB-related symptoms are investigated and treated if diagnosed with TB. We sought to evaluate outcomes of intensified case finding and factors associated with undesirable screening for TB in a large HIV programme in western Kenya.

**Methods:**

We conducted a retrospective study using routine programme data from the AMPATH HIV electronic medical records database for PLHIV in care between 2015 and 2016. Screening for TB was assessed by the recorded presence of cough ≥2 weeks, fever, night sweats, unintentional weight loss, chest pain and/or breathlessness. Undesirable screening was defined as being screened in < 90% of patient clinical encounters. Data were analyzed by encounters and per-patient. Factors associated with undesirable screening were analyzed using log-binomial regression and presented as relative risks.

**Results:**

There were 90,454 PLHIV, 65% females, median age 40 years, median follow-up time of 1.5 years. Total encounters were 683,898, of which screening for TB was recorded in 87%. 1424 (1.6%) PLHIV were not screened at all during the study period. 44% (95% CI: 43.6–44.3) of PLHIV were screened in < 90% of their clinical encounters (undesirable screening). TB-related symptoms were reported in 0.7% of screened encounters, while in 96% of PLHIV, no symptoms were reported. Overall, in 8% of symptomatic encounters sputum microscopy and/or chest radiography results were recorded. 92% of PLHIV did not have TB-related laboratory results recorded for all their symptomatic encounters. Factors which increased the risks of undesirable screening included: attendance at paediatric clinics (aRR: 1.27, 95% CI: 1.20–1.34), being on antiretroviral therapy (aRR: 1.16, 95% CI: 1.13–1.18), having more clinical encounters (aRR: 1.04, 95% CI: 1.04–1.04), and higher patient volumes in a clinic.

**Conclusions:**

There were missed opportunities for screening and testing for TB. Screening was reduced by being on ART, having increased patient-encounters, the clinic setup, and by high patient volumes. HIV programmes should focus on quality of TB care in HIV clinics.

## Background

In 2016 there were an estimated 10.4 million incident tuberculosis (TB) cases worldwide, 10% of whom were people living with HIV (PLHIV) [1]. About 1.7 million deaths were attributed to TB, with more than 374,000 of these occurring among PLHIV. About one-third of deaths among PLHIV are attributed to TB [2].

Global TB incidence rates have been falling since the year 2000. However, this decline has been slow, estimated at 1–2% per annum. If the decline in incidence continues at this rate it will not be possible to reach the 80% reduction in TB incidence that is necessary for meeting the End TB Strategy target and the Sustainable Development Goal of Ending the TB epidemic by 2030 [3].

Kenya is among the top 14 countries worldwide burdened by TB, multidrug-resistant TB (MDR-TB) and TB/HIV [1]. With 1.6 million PLHIV, Kenya is also among the top countries globally with the highest numbers of HIV-infected individuals [4]. A recent nationwide prevalence survey showed that there were an estimated 169,000 incident TB cases in the country in 2016 [1], 17% of which were among PLHIV. However, the country notified only 77,376 cases [1], representing a gap of about 54% who were either undiagnosed or unreported to the national TB programme. This has necessitated a strategic shift in focus to ‘finding the missing cases’.

HIV infection increases the risk of TB disease at least 20-fold [2]. The risk remains substantially elevated even after immune reconstitution with antiretroviral therapy [5]. There is thus need for a collaborative approach to identify and manage TB in this population. One of the pillars of such an approach is intensified case finding (ICF) [6]. ICF can be defined as “the systematic identification of people with presumptive active TB, in a predetermined target group, using tests, examinations or other procedures that can be applied rapidly” [7]. The key to effective ICF is the systematic application of the active screening for TB disease in the predetermined high-risk group by the health workers rather than only screening in response to a specific request or complaint by an individual seeking care. The latter (termed passive case finding) has consistently resulted in over three million infectious TB cases per year being missed by the health system [8]. In a variety of settings ICF strategies, coupled with other routine TB control activities, have been used to reduce TB prevalence, incidence and mortality [9].

With respect to HIV, ICF is the systematic screening for tuberculosis at every encounter in those infected with HIV, are at high risk of HIV, or live in congregate settings [10]. Although different screening strategies yield varying results in different groups of people [8, 10–17], in PLHIV, ICF can result in a high yield with the number needed to screen to identify one case of TB being 10 in high TB incidence countries [12].

PLHIV should be routinely and systematically screened for TB at every clinical encounter using a World Health Organization-approved four-symptom questionnaire inquiring of the presence of cough of any duration, fever, weight loss, and night sweats [18]. A positive response – defined as the presence of one or more of these symptoms – should lead to further confirmatory diagnostic evaluation for TB by Xpert MTB/RIF (*Cepheid, CA, USA*) [19–21]. Other possible investigations include sputum smear microscopy, culture for *Mycobacterium tuberculosis* and chest radiography [22–24]. Those found by testing to have TB are initiated on appropriate TB therapy while those without TB may be considered for isoniazid preventive therapy [25].

In 2014, only seven million PLHIV were reported to have been screened for TB globally [26], a dismal figure compared to the burden of HIV. Whenever screening for TB is done, quality may not always be guaranteed. Given that mortality among PLHIV during TB treatment is high – and mostly occurs within the first two months – missed and delayed diagnosis play contributory roles [27–29]. While different screening and diagnostic strategies vary in their sensitivities and specificities, [18, 28, 29] the applications of those which are especially human-dependent (like symptom screening questioning) are more subjective. Responses to such questions may depend on language, how they are framed, patient current psychological status and existing rapport between staff and the patient. Further, about 15–25% of PLHIV with bacteriologically-confirmed TB disease may be asymptomatic [30]. Other system- and patient-related aspects may also play a significant role.

Although it is part of routine programme performance indicators, ICF among PLHIV has not been systematically evaluated in the programme setting in Kenya. The quality of the screening process has also not been evaluated in large programmes in Kenya. Since being screened for TB does not necessarily guarantee completion of the TB diagnostic pathway, evaluation of the downstream processes are also important.

This study therefore aimed to evaluate, over a period of two years from 2015 to 2016 in a large HIV programme in Kenya; i) the screening for TB among PLHIV by encounters and patients, ii) diagnostic evaluation of identified presumptive TB cases, and iii) factors associated with PLHIV not being screened for TB during their clinical encounters.

## Methods

### Study design

This was a retrospective analysis of routine programme data from the AMPATH electronic Medical Record System (AMRS) database.

### Setting

#### General setting

Kenya is a lower-middle-income country with a population of 48 million, 74% of whom reside in rural areas [31]. The crude death rate is eight per 1000 population per year and life expectancy is about 60 years [32, 33]. HIV prevalence among adults in Kenya is estimated at 5.9% and is higher in western Kenya [34]. Kenya’s Gross Domestic Product stands at USD 70.5 billion with a largely service and agricultural economy [31]. Fourty-6 % of the population still live below the poverty line [35].

The national TB programme carries out various activities aimed at improving TB care, prevention and control in the country. These include case finding approaches targeting both drug sensitive and drug resistant TB patients, children and other special populations, TB/HIV integrated approaches including HIV testing, early co-trimoxazole and antiretroviral therapy (ART) uptake, TB preventive therapy with isoniazid, public private partnerships, laboratory diagnostics, among others. In 2016, the country notified a total of 77,376 TB patients, 31% of whom were co-infected with HIV [1]. A total of 445 drug resistant TB cases were notified in the same year [36]. Treatment success rate was 87 and 82% amongst all notified drug sensitive TB patients and HIV co-infected TB patients respectively while it was 72% among those started on second-line treatment in 2014. Mortality from HIV-positive TB was 50 per 100,000 population in 2016, and ART uptake among TB/HIV co-infected patients was reported to be 95% [1]. In 2014, about 0.5 million PLHIV were reported to have been screened for TB [26].

#### TB/HIV care at AMPATH

The Academic Model Providing Access to Healthcare (AMPATH) programme supports 29 subcounties within eight counties in western Kenya. These facilities have their TB and HIV care and prevention services integrated at various levels [37]. These include screening for TB among PLHIV and HIV testing among TB patients, co-trimoxazole and ART uptake among those co-infected, TB treatment, TB preventive therapy and infection prevention and control. In the fully integrated HIV-TB care set ups, patients are clinically reviewed for both diseases by the same clinical officers (equivalent of physician assistants) with difficult cases handled by medical officers and consultants. The team also has the support of pharmaceutical technologists or pharmacists, counsellors, nutritionists, social workers, records officers and laboratory staff. In the minimally or non-integrated TB-HIV set ups, patients are reviewed for their TB status by nurses and HIV status by clinical officers in different settings or facilities. Difficult cases are referred to higher facilities.

In 2016, over 6000 TB patients were notified in 244 TB or TB/HIV treatment facilities in these supported regions. About one-third of the TB patients were co-infected with HIV. ART and co-trimoxazole therapy uptakes were high at over 95%. Tuberculosis treatment success rate was 88% overall and 83% among HIV positive TB patients.

The AMPATH HIV programme has over 80,000 PLHIV in care at any particular time, of whom about 90% are in 34 of the 140 HIV clinics. The AMPATH electronic Medical Record System (AMRS) [38, 39] – based on Open MRS platform (*OpenMRS, Inc. USA*) – is used to capture patients’ clinical details for majority of the PLHIV. A few clinics still rely fully on paper-based recording systems. However, all the high and medium volume clinics are supported by an AMRS system. Typically, clinicians enter patients details into structured paper-based encounter forms after which the data entry officers transfer the data into the AMRS electronic database. Appropriate queries are used to extract details from the database.

The Kenya national TB/HIV guidelines require that every PLHIV is screened for TB at every clinical visit/ encounter. The screening questions include the presence of cough of any duration, unintentional weight loss, fever, and night sweats [18]. Any affirmative response leads to testing for TB using Xpert MTB/RIF (preferably, if available) or sputum smear microscopy for acid-fast bacilli where Xpert is not available. Any positive test result leads to the diagnosis of TB and subsequent initiation of TB therapy. Those screening negative are offered isoniazid preventive therapy if eligible. Those screening positive but testing negative for TB are investigated further by, for example, chest radiography or given antibiotics and reviewed at subsequent visits. The pulmonary form of TB may still be diagnosed clinically if sputum microscopy and/or Xpert are negative but the chest radiograph or clinical symptoms point to TB disease [19]. Extra-pulmonary TB is usually diagnosed on the basis of clinical presentation though some may access histology (depending on the anatomical site affected) thus strengthening the clinical diagnosis.

During the study period, Xpert MTB/RIF machines were still limited in the study area. Only five sites had resident Xpert machines with some of the remaining sites networked with these in a less-than-optimal systems of sample transport, results relay and recording. As a result Xpert uptake among PLHIV was still low in the setting and when performed results did not get recorded in the HIV database. In contrast, more sites had X-ray capabilities while microscopy services were the most common diagnostics available in the sites. HIV and TB treatment services, Xpert and microscopy are provided free of charge in the country’s public sector. In addition, Xray services for PLHIV are also free for PLHIV in the AMPATH-supported sites.

### Study population

The study population consisted of all PLHIV in the AMRS database and who were in care in the AMPATH programme between January 2015 – December 2016. For programmatic and logistical reasons, all patients in the AMRS were included. Non-clinical encounters such as food and social support were excluded from analysis.

### Data and analysis

Study variables included patient demographics (e.g. gender, age, clinic, encounter type [e.g. youth]) and clinical details (e.g. use of ART, TB screening status and the presence of TB-suggestive symptoms, TB testing results [sputum smear microscopy and chest radiograph] and dates of clinical encounters, screening, and TB test results). Xpert MTB/RIF testing procedures were dropped from the analysis due to the lack of data in the database. Data on whether the PLHIV were on TB therapy during the encounters were also dropped due to significant erroneous recording.

Screening for TB during a clinical encounter was defined as the recorded presence or absence of individual symptoms (cough ≥2 weeks, fever, night sweats, significant weight loss, chest pain and/or breathlessness) by documented checking of the appropriate box; when no ticking was recorded, the encounter was regarded as ‘no screening’. A patient was regarded to have had ‘Undesirable’ screening if s/he had been screened for TB in < 90% of the clinical encounters which the patient had experienced during the study period; otherwise, the patient was categorized as having had ‘Desirable’ screening (i.e. if screened ≥90% of the times in the programme in the study period). The cut-off of ≥90% for desirable screening was chosen as this is also a programmatic target in the country.

At the basic level each observation for each patient was assessed for TB screening and scored as Yes/No. These were then aggregated to the total number of times a patient was screened and divided by the total encounters the patient had to generate the proportion of times the patient was screened for TB. Clinics were categorized as high, medium, low and very low volume based on the number of patients enrolled in the clinics and ensuring equal numbers of clinics per subcategory.

The data were analyzed per-encounter/ observation and per-patient as necessary. Categorical data have been presented in frequencies and proportions and compared using the chi-square test while means (standard deviation) or medians (interquartile range) have been used for continuous variables depending on normality of the data. Any comparisons of continuous variables were by the t-test or mann whitney U test, as appropriate.

Factors influencing the main outcome measure (Undesirable screening) were assessed by log-binomial regression at both bi- and multivariable levels and effects presented as relative risks and their 95% confidence levels. The multivariable model was built sequentially by adding age and gender (selected a priori) and factors identified at bivariable level while assessing confounding, interactions and improvement of the model fit to the data. Variables were also dropped if collinearity or sparsity existed. The final regression model included over 92% of the PLHIV, the difference mainly due to the 8% of PLHIV who did not have data on use of ART. This did not significantly affect the fit of the model. Grossly missing or inaccurate data (Xpert uptake and TB therapy) were not included in the analyses. Level of significance during the analyses was set at *P* < 0.05.

Analyses were carried out using Stata/SE 14.1 software (*StataCorp, College Station, TX, USA*).

## Results

### Patient characteristics

Table 1 shows patient characteristics. There was a total of 90,454 PLHIV in the two-year study period with a median follow up time of 1.5 years (interquartile range, IQR: 34–91 weeks). The data were from 57 health facilities with a median 327 PLHIV per facility (IQR: 89–1634, Range: 7–18,256). Most of the PLHIV (82.8%) were receiving care in the high volume clinics. Of all the PLHIV, 80416 (88.9%) were in care in year 2015 while the rest were newly encountered/ enrolled in 2016.

**Table 1:** Characteristics of the PLHIV enrolled in the AMPATH programme, Kenya (2015–2016)

Characteristics	Total		Females		Males	
	N	(%)	N	(%)	N	(%)
Total	90,454	(100)	59,027	(65.3)	31,427	(34.7)
Year enrolled in care						
Before or in 2015	80,416	(88.9)	52,936	(89.7)	27,480	(87.4)
2016	10,038	(11.1)	6091	(10.3)	3947	(12.6)
Age (years)						
Median [IQR]	40	[29–49]	39	[30–47]	41	[24–51]
Range		[1–87]		[1–87]		[1–87]
0–4	6086	(6.7)	3148	(5.3)	2938	(9.4)
5–9	3442	(3.8)	1769	(3.0)	1673	(5.3)
10–19	5652	(6.3)	3031	(5.1)	2621	(8.3)
20–24	2750	(3.0)	2056	(3.5)	694	(2.2)
25–34	14,725	(16.3)	11,634	(19.7)	3091	(9.8)
35–44	25,836	(28.6)	18,236	(30.9)	7600	(24.2)
45–54	19,892	(22.0)	12,400	(21.0)	7492	(23.8)
55–64	9394	(10.4)	5352	(9.1)	4042	(12.9)
65+	2664	(3.0)	1394	(2.4)	1270	(4.0)
Clinic category						
Paediatric clinics	13,978	(15.5)	7227	(12.2)	6751	(21.5)
Youth clinics	383	(0.4)	207	(0.4)	176	(0.6)
PMTCT clinics	4328	(4.8)	4328	(7.3)	–	
Adults clinics	71,765	(79.3)	47,293	(80.1)	24,472	(77.9)
Uptake of ART						
Not on ART	15,302	(18.3)	9282	(16.9)	6020	(20.9)
On ART	68,366	(81.7)	45,642	(83.1)	22,724	(79.1)
*Missing*	*6786*		*4103*		*2683*	
Clinic volume^a^						
High volume (pts)	74,900	(82.8)	48,715	(82.5)	26,185	(83.3)
Medium volume (pts)	12,687	(14.0)	8449	(14.3)	4238	(13.5)
Low volume (pts)	2322	(2.6)	1539	(2.6)	783	(2.5)
Very low volume (pts)	545	(0.6)	324	(0.6)	221	(0.7)

*PLHIV* – People Living with HIV, *IQR* – Interquartile range, *PMTCT* – prevention of mother to child transmission, *ART* – antiretroviral therapy, *pts*. – total patients

^a^High volume clinics – had 1634+ PLHIV, Medium volume – 327 to 1633 PLHIV, Low volume – 89 to 326 PLHIV, Very low volume – < 89 PLHIV, based on equal number of clinics per subcategory

Of all the PLHIV 59027 (65.3%) were females and the median age was 40 years (IQR: 29–49). Youth and paediatric clinics were attended by 0.4 and 15.5% of the PLHIV, respectively. At the time of analysis, 68,366 (81.7%) of PLHIV were on ART. The variable ‘on TB treatment’ was dropped from the analysis because of significant erroneous recording.

### Screening for TB

**Fig. 1:**
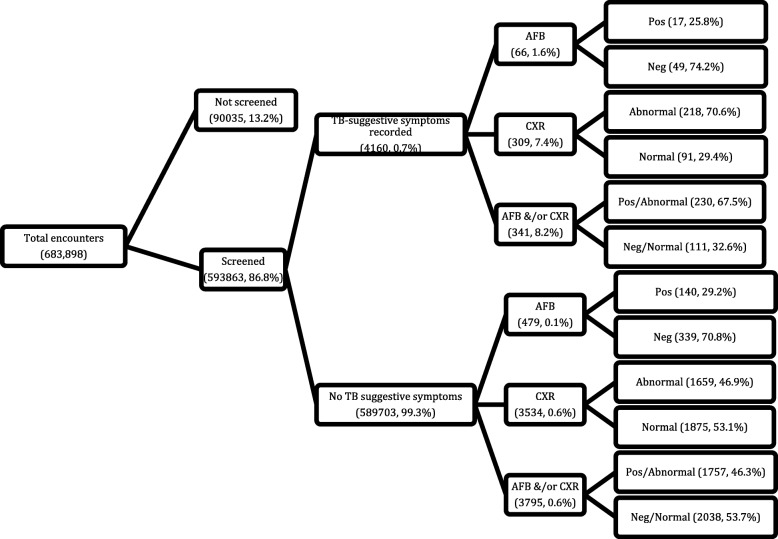
Screening and diagnostic cascade of the HIV clinical encounters in the AMPATH programme, Kenya (2015–2016). AFB – sputum smear microscopy (Acid fast bacilli); CXR – chest radiograph; Pos – positive for AFBs; Neg – negative for AFBs

**Fig. 2:**
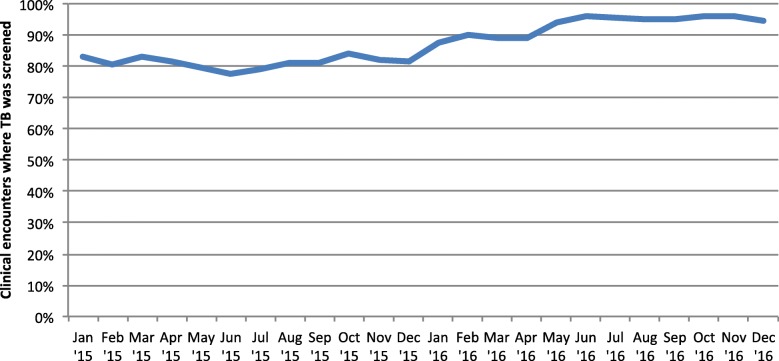
Monthly tuberculosis screening trends among HIV clinical encounters, AMPATH programme, 2015–2016

Analyzing the data by clinical encounters – *and not by patients* – showed that there were 683,898 clinical encounters in the two years. Screening for TB was carried out in 593,863 (86.8%) of the clinical encounters (Fig. 1). Screening averaged 87.1% per month (standard deviation, SD: 6.6%) and increased gradually during the period – from 83% in January 2015 to 94% in December 2016 (*P*-value for trend < 0.001) (Fig. 2).

Median number of encounters per PLHIV was 8 (IQR: 4–10). Average screening per patient was 87.2% (SD: 18.9) of the encounters. A total of 1424 PLHIV (1.6%) were not screened at all for TB during the two-year period while 89,030 (98.4%) of the PLHIV were screened at least once. 46,554 (51.5%) of the PLHIV were screened for TB in all their clinical encounters.

A total of 39,773 (44.0% [95% confidence interval, CI: 43.6–44.3]) PLHIV were screened for TB in < 90% of their clinical encounters (Undesirable screening) within the programme in the period of study. The remainder (50,681 [56.0%]) were screened in ≥90% of their clinical encounters (Desirable screening).

**Fig. 3:**
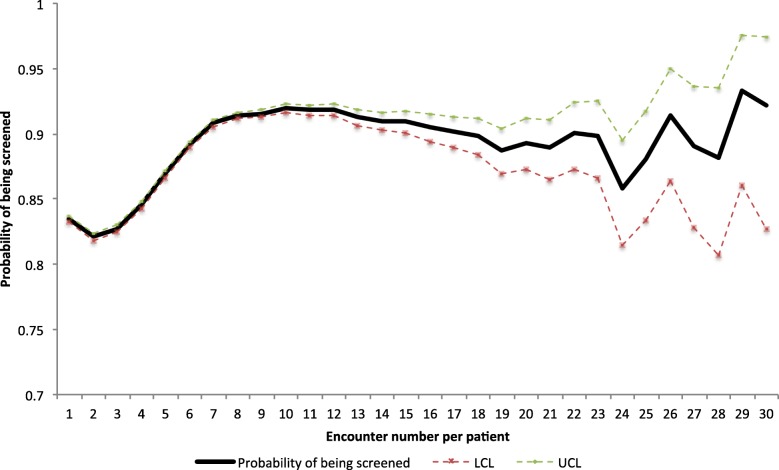
Probability of being screened for tuberculosis among PLHIV enrolled in the AMPATH programme, Kenya (2015–2016). LCL – lower limit; and UCL – upper limit of 95% confidence interval

The probability of PLHIV being screened in their first clinical encounter in the programme was 83.5% (95% CI: 83.2–83.7) (Fig. 3). The probability of being screened increased to a peak of 91.9% by the 10th clinical encounter, then decreased progressively till the 19th encounter. The probability of being screened then fluctuated afterwards and with wide confidence intervals (likely due to low numbers of PLHIV with such visits).

### Presence of symptoms suggestive of TB

Of the encounters in which TB screening was carried out, the presence of any symptom suggestive of TB was reported in 4160 (0.7%) of the clinical encounters (Fig. 1). Cough ≥2 weeks was the most common TB-suggestive symptom occurring in 69.9% of the encounters in which symptoms were reported, followed by chest pain (23.0%), noticeable weight loss (16.6%), night sweats (16.2%), breathlessness (15.1%), and lastly fever (12.4%). Among the symptomatic encounters, cough and chest pain were the most frequent combination of symptoms.

In 86,819 (96.0%) of the PLHIV, no symptoms suggestive of TB were ever reported during the study period. Another 3225 (3.6%) of the PLHIV reported symptoms suggestive of TB in only one clinical encounter. The remainder of the PLHIV (410 [0.4%]) reported symptoms of TB in more than one clinical encounter (up to eight encounters). A total of 354 (0.4%) of the PLHIV reported symptoms of TB in all their encounters in which TB screening was done.

### Testing and diagnosis of TB (using sputum microscopy and chest radiographs)

Of the 4160 symptomatic encounters, only 66 (1.6%) and 309 (7.4%) had sputum smear and CXR results indicated, respectively (Fig. 1). Of these, 17 (25.8%) were positive for AFB and 218 (70.6%) had an abnormal CXR. Overall, 341 (8.2%) of the symptomatic encounters had either AFB and/or CXR results recorded, of which 230 (67.4%) were positive/ abnormal. Of the 589,703 encounters where no TB suggestive symptoms were recorded, 3795 (0.6%) also had AFB and/or CXR results, 1757 (46.3%) of which were positive/ abnormal.

A total of 3332 (91.6%) PLHIV did not have AFB or CXR results recorded for all the encounters during which they were symptomatic during the study period while 261 (7.2%) had AFB and/or CXR results recorded for all the encounters during which they were symptomatic. The remainder (42 [1.2%]) had AFB and/or CXR results variably recorded when they were symptomatic.

### Factors associated with undesirable screening for TB

Fourty-4 % of the PLHIV had undesirable screening for TB. This did not differ between males and females at bivariate level and after adjusting for co-variates (Table 2). When compared to adults aged 35–44 years, the younger age groups generally had lower risk of undesirable screening while the older age groups had increased risk.

**Table 2:** Factors associated with undesirable screening^a^ for tuberculosis among PLHIV in the AMPATH programme, Kenya (2015–2016)

Variable	Total	Undesirable Screening^a^		cRR (95% CI)	aRR (95% CI)
	N	N	(%)		
Gender					
Female	59,027	25,827	(43.8)	1	1
Male	31,427	13,946	(44.4)	1.01 (0.99–1.03)	0.99 (0.98–1.01)
Age (years)					
35–44	25,836	11,058	(42.8)	1	1
0–4	6086	2363	(38.8)	0.91 (0.88–0.94)	0.93 (0.87–0.99)
5–9	3442	1691	(49.1)	1.15 (1.11–1.19)	0.99 (0.93–1.05)
10–19	5652	3355	(59.4)	1.39 (1.35–1.42)	1.05 (1.00–1.11)
20–24	2750	966	(35.1)	0.82 (0.78–0.87)	0.92 (0.87–0.96)
25–34	14,725	5309	(36.1)	0.84 (0.82–0.86)	0.93 (0.90–0.95)
45–54	19,892	9452	(47.5)	1.11 (1.09–1.13)	1.07 (1.05–1.09)
55–64	9394	4364	(46.5)	1.09 (1.06–1.11)	1.04 (1.01–1.06)
65+	2664	1211	(45.5)	1.06 (1.02–1.11)	1.00 (0.96–1.05)
Clinic category					
Adults clinics	71,765	31,126	(43.4)	1	1
Paediatric clinics	13,978	6923	(49.5)	1.14 (1.12–1.16)	1.27 (1.20–1.34)
Youth clinics	383	126	(32.9)	0.76 (0.66–0.88)	0.80 (0.67–0.94)
PMTCT clinics	4328	1598	(36.9)	0.85 (0.82–0.89)	0.89 (0.85–0.92)
Uptake of ART					
Not on ART	15,302	5979	(39.1)	1	1
On ART	68,366	32,541	(47.6)	1.22 (1.19–1.24)	1.16 (1.13–1.18)
Total encounters				1.05 (1.04–1.05)	1.04 (1.04–1.04)
Clinic volume^b^					
High volume (pts)	74,900	33,334	(44.5)	1	1
Medium volume (pts)	12,687	5415	(42.7)	0.96 (0.94–0.98)	0.92 (0.90–0.94)
Low volume (pts)	2322	839	(36.1)	0.81 (0.77–0.86)	0.85 (0.80–0.89)
Very low volume (pts)	545	185	(33.9)	0.76 (0.68–0.86)	0.94 (0.84–1.04)

*cRR* – crude/unadjusted relative risk, *aRR* – adjusted relative risk, *CI* – Confidence, *PLHIV* – People Living with HIV, *PMTCT* – prevention of mother to child transmission, *ART* – antiretroviral therapy, pts. – total patients

^a^Screening defined as ‘Undesirable’ if a patient was recorded to have been screened for tuberculosis in < 90% of his/her clinical encounters

^b^High volume clinics – had 1634+ PLHIV; Medium volume – 327 to 1633 PLHIV; Low volume – 89 to 326 PLHIV; Very low volume – < 89 PLHIV; based on equal number of clinics per subcategory

Compared to those visiting adult clinics, PLHIV seen at the PMTCT and youth clinics had 11 and 20% lower risk of undesirable screening, respectively while those in the paediatric clinics had 27% increased risk of undesirable screening. Being on ART was associated with a 16% increased risk of undesirable screening for TB. A unit increase in the number of clinical encounters a patient had resulted into 4% increased risk of undesirable screening. Higher patient volumes in the clinics increased the risk of undesirable screening.

## Discussion

In this study, slightly more than half of the PLHIV had documented screening for TB in all their clinical encounters. When screening was documented symptoms were reported in less than 1% of encounters and 4% of PLHIV during the study period. Among those with recorded symptoms less than one-in-ten had sputum microscopy or CXR results documented. Being on ART, having more clinical encounters, high patient volumes in a clinic and attendance at paediatric clinics increased the risk of not being screened.

The study had several strengths: it followed up a large number of patients over a considerable duration of time and the assessment was based on data obtained from the routine programme setting giving a realistic picture of the situation on the ground. These make the results easily generalizable at the programmatic level.

To ‘find the missing cases’ screening for TB should be systematically conducted in all the high-risk populations, including PLHIV. Those screening positive for any of the symptoms should be tested using appropriate tools while those diagnosed with TB be promptly initiated on appropriate TB therapy. Missed opportunities in this care cascade have been reported in other settings as well. A recent study in South Africa reported that 39% of patients with TB were not screened at the primary care setting, and among those screened, 62% were not tested for TB [40]. These screening rates were comparable to those of our study which also reported far less testing – < 10% of the symptomatic. In Mozambique, 61% of a nationally-representative sample of patients newly initiating ART were screened for TB [41]. However, this varied from 2 to 98% according to the setting. In the same study, similar to the current study, screening rates increased over the years.

The World Health Organization advises that if screening is carried out properly, between 10 and 20% of PLHIV report at least one symptom suggestive of TB [21]. The < 1% and overall 4% positive symptom screen in clinical encounters and PLHIV reported in our study, respectively is thus far lower than that expected in such a setting. Conversely, a study in a hospital HIV clinic in Ethiopia reported higher positive symptom screen at 39% [42].

The identified factors associated with higher risk of undesirable screening like being on ART, more clinical encounters/visits per patient, higher patient volume in a clinic, and paediatric clinic setup are likely more than just patient-related; they are also system- and health worker-related. A clinician is likely to spend more effort and time managing adherence and medication related issues for a patient on ART than focus on screening for TB. While some clinicians are aware that ART reduces the risk of TB disease, this is unlikely to play a major role in the lack of screening in this setting.

More visits by a patient to the clinic slightly reduced the chances of being screened for TB. While it is expected that more clinical encounters/ visits by a patient may lead to familiarity with the clinicians and the system, it has the unintended risk of skipping some processes (for example, TB screening or recording) especially if not regularly emphasized. Increased patient volume ultimately results in less time spent per patient thus affecting quality of care especially if the number of clinicians does not increase in tandem. The challenges associated with screening and diagnosis of TB in the paediatric age-group may explain the reduced screening in this subgroup.

The findings in this study have several implications: First, the study reports poor screening and testing for TB despite having structured clinical encounter forms with specific checks for TB screening, TB-related diagnostics and other aspects of care. Similar findings were reported by a clinical audit in a large UK urban clinic which found that only 6–12% of routine tuberculosis screening occurred among patients enrolled in the HIV programme despite implementing screening prompts [43]. Thus there is need for a systematic audit of the vast amount of data being generated by the AMPATH HIV programme. Such an audit should assess, but not limit itself to, i) the quality of screening for TB in the programme, ii) access to TB diagnostics including Xpert, iii) the recording processes, and iv) the overall quality of TB data in the database.

Second, there is need to strengthen the weaker links in TB/HIV integration which enhance screening and testing for TB, including documentation. These include fully adopting the ‘two diseases, one patient, one clinic, one appointment, one health worker’ approach [37]. Such an approach helps avoid patient juggling, increases knowledge and expertise of health workers in managing both diseases and improves documentation.

Third, there is need to strengthen sputum transportation system and access to Xpert testing platform including timely result feedback. Specimen transportation should be customized to the individual site characteristics and can include the use of motorcycle transporters and/ or health workers. Result relay can be further strengthened by optimizing the use of technology to transmit the results, for example, automated SMS platforms. Commodity supply and logistics must also be streamlined for full functionality.

Finally, HIV programmes should shift their focus to also include quality of the screening and attendant processes among the PLHIV. This can include regular audits as aforementioned and qualitative reviews.

The study had some limitations. We report on few co-variates likely affecting adequate adjustments for confounders. We do not, in particular, report nor adjust for direct provider-related factors like attitude and knowledge. Another limitation is the non-independence and clustering of data analyzed at encounter/ observation level. We, however, also describe the data per-patient as necessary. The binary cut-off of < 90% for undesirable screening though based on programmatic targets, is arbitrary and outcome may depend on duration of evaluation/ follow up. Finally, as a retrospective study, there were significant challenges to the data that included missing and/or inconsistent data on Xpert uptake and TB treatment – which are key to evaluating any TB/HIV programme. It is thus possible that some of our findings could be attributed to poor documentation and reporting bias.

## Conclusion

This study found slightly more than half of PLHIV having documented screening for TB and low symptom and TB-testing recording. ART, more encounters, high patient volumes, and paediatric clinic setup reduced screening rates. Programmes should strengthen the ICF process for PLHIV and focus on the quality of the screening and recording of TB in the HIV clinics.

## Abbreviations

AFB: Acid fast bacilli
AMPATH: Academic Model Providing Access to Healthcare
AMRS: AMPATH electronic Medical Record System
ART: antiretroviral therapy
CI: Confidence interval
CXR: Chest radiograph
HIV: Human Immunodeficiency Virus
ICF: Intensified case finding
IQR: Interquartile range
LAM: Lipoarabinomannan antigen urine test
MDR-TB: Multidrug resistant tuberculosis
PLHIV: People Living with HIV
PMTCT: Prevention of mother to child transmission
RR: Relative risk
TB: Tuberculosis
